# Depression, not PTSD, is associated with attentional biases for emotional visual cues in early traumatized individuals with PTSD

**DOI:** 10.3389/fpsyg.2014.01474

**Published:** 2015-01-06

**Authors:** Charlotte E. Wittekind, Christoph Muhtz, Lena Jelinek, Steffen Moritz

**Affiliations:** ^1^Clinical Neuropsychology, Department of Psychiatry and Psychotherapy, University Medical Center Hamburg-EppendorfHamburg, Germany; ^2^Department of Psychosomatic Medicine, University Medical Center Hamburg-EppendorfHamburg, Germany; ^3^Schön Klinik Hamburg-EilbekHamburg, Germany; ^4^Department of Psychiatry and Psychotherapy, University Medical Center Hamburg-EppendorfHamburg, Germany

**Keywords:** attentional bias, trauma, depression, bias components, transmission

## Abstract

Using variants of the emotional Stroop task (EST), a large number of studies demonstrated attentional biases in individuals with PTSD across different types of trauma. However, the specificity and robustness of the emotional Stroop effect in PTSD have been questioned recently. In particular, the paradigm cannot disentangle underlying cognitive mechanisms. Transgenerational studies provide evidence that consequences of trauma are not limited to the traumatized people, but extend to close relatives, especially the children. To further investigate attentional biases in PTSD and to shed light on the underlying cognitive mechanism(s), a spatial-cueing paradigm with pictures of different emotional valence (neutral, anxiety, depression, trauma) was administered to individuals displaced as children during World War II (WWII) with (*n* = 22) and without PTSD (*n* = 26) as well as to non-traumatized controls (*n* = 22). To assess whether parental PTSD is associated with biased information processing in children, each one adult offspring was also included in the study. PTSD was not associated with attentional biases for trauma-related stimuli. There was no evidence for a transgenerational transmission of biased information processing. However, when samples were regrouped based on current depression, a reduced inhibition of return (IOR) effect emerged for depression-related cues. IOR refers to the phenomenon that with longer intervals between cue and target the validity effect is reversed: uncued locations are associated with shorter and cued locations with longer RTs. The results diverge from EST studies and demonstrate that findings on attentional biases yield equivocal results across different paradigms. Attentional biases for trauma-related material may only appear for verbal but not for visual stimuli in an elderly population with childhood trauma with PTSD. Future studies should more closely investigate whether findings from younger trauma populations also manifest in older trauma survivors.

## Introduction

In 2014, the United Nations Refugee Agency (UNHCR; United Nations Refugee Agency, 2014) reported that at the end of 2013, more than 50 million people were forcibly displaced due to war, conflicts, or human right violations. The negative consequences of forced displacement for the psychological and physical well-being are documented in many studies (e.g., Fazel et al., 2005; Porter and Haslam, 2005). Beyond that, forced displacement is associated with a highly increased risk for posttraumatic stress disorder (PTSD, e.g., Fazel et al., 2005; Steel et al., 2009; Freitag et al., 2013). Even decades later, a substantial proportion of those affected suffer from negative consequences (e.g., Muhtz et al., 2011; Freitag et al., 2013). In addition, the negative consequences of traumatic events are not restricted to those directly exposed, but seem to impact close relatives such as the children (Danieli, 1998; Leen-Feldner et al., 2013). For example, an increased risk for the development of PTSD was reported in offspring of individuals with PTSD (Baider et al., 2000, 2006; Yehuda et al., 2008). Nonetheless, the literature is not fully consistent and adequately designed population-based studies did not find clear-cut evidence for a transgenerational transmission (Van IJzendoorn et al., 2003; Levav et al., 2007; Fridman et al., 2011).

In order to study the long-term consequences of forced displacement, individuals displaced at the end of World War II (WWII) were increasingly investigated in recent years. Studies revealed a high rate of PTSD more than 60 years later (e.g., Teegen and Meister, 2000; Muhtz et al., 2011). According to DSM-IV (American Psychiatric Association [APA], 2000), PTSD is characterized by symptoms of re-experiencing, avoidance of trauma-related stimuli, and hyperarousal. Beyond that, PTSD is associated with different biases in information processing, for example, attentional biases for trauma-related stimuli (Buckley et al., 2000; Constans, 2005).

In experimental psychopathology, different paradigms have been adopted from cognitive psychology to investigate attentional biases in PTSD, most commonly the emotional Stroop task (EST, Williams et al., 1996) and the dot-probe paradigm (MacLeod et al., 1986). EST studies largely contributed to our knowledge of attentional biases in PTSD and the emotional Stroop effect (ESE) was considered a robust finding for many years as it was replicated across different trauma populations (for reviews see Buckley et al., 2000; Constans, 2005). However, the specificity and stability of the effect were critically discussed (Kimble et al., 2009; Cisler et al., 2011) and closer scrutiny reveals that interpretability of many studies is constrained by methodological limitations. For example, while some studies lack a trauma control group (Paunovic et al., 2002; El Khoury-Malhame et al., 2011a; Fleurkens et al., 2011), the interpretability of other studies is restricted because no negatively-valenced control stimuli were included (Harvey et al., 1996; Paunovic et al., 2002; Bremner et al., 2004). Thus, the question whether the effect is specific to PTSD and/or to trauma-related stimuli remains unresolved. Furthermore, the EST has several methodological problems (e.g., Algom et al., 2004) and does not allow to disentangle the different attentional bias components, that is, whether these are comprised of *attentional facilitation* (i.e., preferred processing of trauma-related compared to neutral stimuli), *attentional interference* (i.e., difficulties disengaging from trauma-related to other stimuli) or *attentional avoidance* (i.e., attention allocation toward the opposite location of trauma-related stimuli, cf. Cisler and Koster, 2010). The differentiation of these components can provide a better theoretical understanding and allows the development or improvement, respectively, of novel interventional techniques (Shipherd and Salters-Pedneault, 2008).

In dot-probe tasks, two stimuli of different valence are presented simultaneously for a set time (e.g., 500 ms). Subsequently, one of the two stimuli is replaced by a probe and participants are asked to respond to either its location or to classify the probe (e.g., ^*^ or ^**^, Cisler et al., 2009). A faster reaction to probes that replace negatively-valenced (congruent trials) compared to neutral stimuli (incongruent trials) are interpreted as evidence for attentional facilitation as attention is already drawn to the spatial location of the threatening stimulus (Yiend, 2010). The opposite response pattern, that is, slower reactions to probes that replace negatively-valenced compared to neutral stimuli are indicative of attentional avoidance (Cisler and Koster, 2010; Yiend, 2010). The majority of studies administering variants of the dot-probe paradigm in PTSD do not provide evidence for attentional biases for trauma-related/negative material (Dalgleish et al., 2003; Elsesser et al., 2004, 2005; Fani et al., 2011); however, some studies demonstrated attentional biases (Bryant and Harvey, 1997; El Khoury-Malhame et al., 2011a,b). More recent studies suggest that acute stress (e.g., missile attacks) is associated with attentional avoidance of threat-related information (Bar-Haim et al., 2010; Wald et al., 2011a,b), which, in turn, predicted psychopathological symptoms in the short- (Bar-Haim et al., 2010; Wald et al., 2011a) and long-term (Wald et al., 2011b).

A paradigm that allows the differentiated assessment of attentional bias components represents the visual search task (VST, Öhmann et al., 2001). In VSTs, participants are asked to detect a discrepant target stimulus embedded in an array of identical stimuli. The VST was applied in two studies to differentially assess facilitation and interference in PTSD (Pineles et al., 2007, 2009). In the interference condition, a target (e.g., non-word) was presented in an array of experimental stimuli (e.g., trauma-related words) whereas in the facilitation condition, the arrangement was reversed (i.e., target experimental word embedded in an array of identical non-words). Facilitation to trauma-related words was inferred from faster reaction times to trauma-related compared to neutral targets in an array of non-word distracters. Interference to trauma-related words was inferred from slower reaction times to target stimuli embedded in arrays with trauma-related compared to neutral distracters (Pineles et al., 2009). In both studies, PTSD was associated with attentional interference to trauma-related stimuli (Pineles et al., 2007, 2009) and this effect was specific to trauma-related stimuli (Pineles et al., 2009). However, there was no evidence for attentional facilitation. This finding conflicts with the theoretical assumption of hypervigilance in PTSD (Pineles et al., 2009). However, the latter two paradigms are also plagued by interpretational problems (see Hauschildt et al., 2013, for a further discussion).

### Spatial-cueing task

One paradigm that enables the assessment of the precise underlying mechanism represents a modification of the *spatial-cueing paradigm* (Posner, 1980). A great advantage of cueing paradigms is the fact that the behavioral reaction is made in response to a neutral target, thus, response bias explanations can be ruled out (Yiend, 2010). Furthermore, by varying the stimulus-onset asynchrony (SOA), cueing paradigms allow the assessment of the temporal attention allocation. This is important when investigating attentional biases in PTSD as this disorder seems to be associated with delayed disengagement from trauma-related cues (e.g., Pineles et al., 2007) and disengagement is based upon controlled processes that need more time to take effect (cf. Yiend, 2010).

Attention is focused on a fixation point located between two rectangles. Subsequently, a cue is presented in one of the two rectangles, followed by a target that either appears in the same rectangle (valid trial) or in the opposite rectangle (invalid trial). In some trials, no cue appears (catch trials). The participants' task is to indicate (e.g., key press) in which rectangle the target was presented. For the assessment of attentional biases in psychopathology, the cue is varied as to its emotional valence (e.g., threatening, neutral). While facilitation is operationalized as faster responses to validly cued trials when the cue is threatening/disorder-specific compared to neutral, avoidance is characterized by slower RTs to threatening compared to neutral cues in valid trials. Slower RTs to threatening/disorder-specific compared to neutral stimuli in invalidly cued trials are interpreted as interference, faster RTs as avoidance (Koster et al., 2006). However, this pattern is only true for short SOAs (<300 ms, Posner and Cohen, 1984). With longer time intervals between cue and target (SOA), *inhibition of return* (IOR) occurs, that is, cued locations lose their attentional preference as attention is directed to uncued locations after a certain time (for a review see Klein, 2000). It is assumed that this effect is adaptive as redirecting attention to an already attended location does not provide additional information. As PTSD seems to be associated with problems in disengaging and patients “stick” to trauma-related material, the IOR effect should be reduced or even absent as attention is not re-directed to new locations. Although some studies provide evidence for this assumption in anxiety (Nelson et al., 1993; Fox et al., 2002), other results speak for the stability of the IOR effect (Stoyanova et al., 2007; Lange et al., 2008). To the best of our knowledge, one study applied the spatial-cueing paradigm in PTSD (Hauschildt et al., 2013). A spatial-cueing paradigm with pictures of different emotional valence (trauma-related, negative control, general threat, neutral) and varying SOA (450, 1200 ms) was administered to 25 participants with PTSD, 22 non-PTSD and 24 healthy control participants. Although neither PTSD nor trauma exposition were associated with attentional biases, depressive symptomatology was linked with attentional avoidance of trauma-related and negative control stimuli.

### Transgenerational information processing studies

First evidence that information processing biases can be transferred came from Motta and colleagues (Motta et al., 1994, 1997) who administered an EST to children of Vietnam veterans and non-veterans. In the first study (Motta et al., 1994) the mean difference between children of veterans and non-veterans for the PTSD-related card was 1.97 s, whereas the mean reaction time differences for all other cards varied between 0.21 and 0.81 s. In a replication study with a larger sample, children of veterans were significantly slower to color-name the war-related card compared to children of non-veterans, whereas RTs to all other cards (OCD-related, positive, neutral) did not differ between groups (Motta et al., 1997). Evidence for a transmission was also found when the children's group allocation was based on parental trauma exposure (Suozzi and Motta, 2004). These findings were replicated in a sample of children and adolescents (Moradi et al., 1999): children whose parents suffered from PTSD exhibited an ESE for threat-related compared to neutral words and compared to the children of healthy control participants. However, conflicting evidence stems from one study in which children of displaced individuals (with and without PTSD) were compared to children of non-traumatized healthy control participants regarding their color naming latencies in an EST (Wittekind et al., 2010). There was no evidence for attentional biases for trauma-related words in children of displaced individuals with PTSD. However, the sample differed from previous studies in several important aspects (e.g., time since parental traumatization, children's age, trauma type, parental trauma vs. PTSD) limiting comparability between studies.

To conclude, a substantial body of studies assert that PTSD is related to attentional biases for trauma-related material which seem to results from difficulties to disengage (Pineles et al., 2007, 2009). However, interpretability of many studies is constrained by methodological limitations and results need to be replicated across different paradigms and stimulus modalities (i.e., verbal vs. visual stimuli). Furthermore, prior research almost exclusively recruited younger trauma samples (average age in emotional Stroop studies: 36 years, Cisler et al., 2011), thus, it remains unclear whether attentional biases persist over the course of the disorder. Beyond that, essential influencing factors (e.g., SOA, stimulus type, comorbid depression) have been neglected in prior research on attentional biases in PTSD (also see Cisler et al., 2009). Transgenerational studies applied the EST to assess whether parental trauma or PTSD, respectively, is related to attentional biases in the second generation. Beside the fact that evidence is ambiguous, it is yet unclear whether findings translate to different paradigms and which attentional bias component drives the effect.

### The present study

The aim of the present study was to replicate and extend the results by Hauschildt et al. (2013) in a sample of older individuals with chronic PTSD due to childhood trauma as well as their offspring. To meet this aim, we also administered a modified version of the spatial-cueing paradigm using visual instead of verbal stimuli that differed as to their emotional valence (trauma-related, depression-related, anxiety-related, neutral). As PTSD seems to be associated with delayed disengagement from trauma-related stimuli (Pineles et al., 2007, 2009), one would expect a diminished IOR effect for targets following trauma-related compared to other emotional or neutral pictures in individuals with PTSD. However, as the majority of previous studies do not provide evidence for a reduced IOR effect in PTSD (Hauschildt et al., 2013) and other anxiety disorders (Stoyanova et al., 2007; Lange et al., 2008), we assume that the IOR effect is not affected by cue valence. Regarding a transgenerational transmission, we hypothesized that offspring of PTSD participants demonstrate an attentional bias for trauma-related material; however, we did not have a directed hypothesis whether attentional biases result from facilitation, interference, or avoidance.

## Materials and methods

### Participants

Individuals displaced as children during or after WWII (*n* = 50) and one of their adult children were recruited by (a) a database built up in a previous study (for a detailed description of recruitment strategies see Muhtz et al., 2011), (b) contact to displacement networks and self-help groups, (c) word of mouth, and (d) personal contacts. Participants were born between 1932 and 1941 and experienced at least one traumatic event according to DSM-IV trauma criteria during their flight. Group allocation was based on the PTSD module of the Structured Clinical Interview for DSM-IV (SCID-I, First et al., 1997). To assure that diagnoses of PTSD were indeed due to forced displacement and not to a later trauma, we inquired whether participants experienced a traumatic event other than flight/displacement. If this was the case, participants had to indicate which of the traumatic events was worse. Subsequently, PTSD criteria for each traumatic event were assessed via the SCID and items were rephrased such that the relation to the respective event was stressed, e.g., instead of “traumatic event” we explicitly used “after displacement.” Exclusion criteria for all groups were a lifetime history of psychotic, manic or bipolar symptoms, substance dependence within the last year or suicidal tendencies as assessed with the MINI Neuropsychiatric Interview (MINI, Sheehan et al., 1998). Of all traumatized participants who were assessed, two participants had to be excluded (manic disorder, trauma criteria A2 not fulfilled). Three adult children were excluded due to alcohol dependence, withdrawal of informed consent, and psychotic symptoms. Thus, the PTSD group comprised 22 traumatized participants of whom 12 fulfilled all PTSD criteria and 10 participants were diagnosed with subsyndromal PTSD as suggested by Blanchard et al. (1996, DSM-IV criteria A, B, E, F were fulfilled and either criterion C or D) and 21 of their adult children. The remaining 26 participants (and 24 of their children) were allocated to the non-PTSD group. Twenty-two non-traumatized (DSM-IV trauma criteria A1 and A2) participants who were not displaced during WWII, not married to an individual displaced during WWII, not meeting any current axis I disorder (based on the MINI) and one adult offspring formed the healthy control group. The latter group was recruited by means of advertisement in local media, notices in public places, and word of mouth. Written informed consent was obtained prior to the study from all participants. The study was approved by the local ethics committee.

### Measures

#### Psychopathology

All participants were interviewed with the MINI interview (Sheehan et al., 1998) in order to determine (a) exclusion criteria for all participants, (b) (comorbid) psychiatric disorders in traumatized participants, and (c) absence of any current axis I disorder in non-traumatized controls. In order to quantify PTSD severity, the Posttraumatic Diagnostic Scale (PDS, Foa et al., 1997) was administered to all traumatized participants. The PDS is a self-report questionnaire showing high reliability and validity (Foa et al., 1997). All 17 items of the PDS were paraphrased such that “traumatic event” was replaced by “flight/displacement.” Depression severity was quantified with the 17-item version of the Hamilton Depression Rating Scale (HDRS, Hamilton, 1960). Finally, verbal intelligence was estimated using a vocabulary test [Mehrfachwahl-Wortschatz-Intelligenztest B (MWT-B), Lehrl, 2005].

#### Stimulus selection

Pictorial stimuli of the present study captured five different conditions (Trauma, Depression, Anxiety, Neutral, Neutral old). Pictures were selected from the *International Affective Picture System* (IAPS, Lang et al., 2008), the internet or from books and media reports about displacement after WWII (trauma-related stimuli). Besides the emotional conditions and the neutral condition (IAPS pictures), we included a fifth condition (neutral old) containing pictures that came from the same time as the trauma- (i.e., displacement) related pictures. This was done to control for “age effects” as it is conceivable that trauma-related pictures are processed differentially due to their deviation from pictures taken from the internet or the IAPS. All stimuli were rated by 15 displaced individuals in a pilot study that was conducted via an online survey regarding (a) their relevance for (aa) flight/displacement after WWII, (ab) depression and (ac) anxiety (1 = very relevant, 2 = slightly relevant, 3 = not relevant), (b) neutrality (yes/no), and (c) personal relevance (yes/no). For the final picture set, trauma-related pictures had to be rated as highly displacement-relevant (rating = very relevant) by at least 80% and as personally relevant by at least 60% of displaced individuals. Furthermore, trauma-related pictures were rated as significantly more displacement-relevant than pictures from all other categories, all *p*s < 0.001. The final set of pictures comprised 10 trauma-related (e.g., refugee trek), 10 depression-related (e.g., sad person), 10 anxiety-related (e.g., snake), 10 neutral (IAPS, e.g., towel), and 10 neutral-old pictures (e.g., landscape). Pictures were presented in black-and-white.

#### Procedure and experimental task

Before the experimental paradigm started, demographic and psychopathological information (MINI, HDRS) were thoroughly inquired. Traumatized participants were also assessed with the PTSD module of the SCID.

The experimental paradigm was constructed using Superlab® software and was presented individually via a Macintosh computer in a dimly lit room to prevent reflections on the monitor. Participants were instructed in written and verbal form to classify via key press whether a target (black dot) was presented in the right or left rectangle (“m” and “y” [German keyboard], respectively, on the keyboard). They were told that each target would be preceded by a picture whose position was irrelevant for the task. To ensure that all participants understood the task, a practice trial with 10 items was administered to participants prior to the experimental task.

The procedure for each trial was as followed: to focus attention to a central point, a small fixation cross was presented between two rectangles (7 cm high by 9.4 cm wide) for 500 ms. The rectangles remained on the screen throughout a block of trials. Subsequently, a cue stimulus picture appeared with equal probability inside one of the two rectangles (400 ms). The cue varied as to its emotional valence (Trauma, Depression, Anxiety, Neutral-old, Neutral) and was followed by the fixation cross/rectangles for either 50 or 800 ms. Thus, SOA between cue and target varied between short (450 ms) and long (1200 ms) intervals (Moritz et al., 2009; Hauschildt et al., 2013). Then, the target was presented equally often in the center of one of the two rectangles and independent of the cue (i.e., the position of the cue had no predictive value for the position of the target). In valid trials, cue and target appeared in the same rectangle, whereas in invalid trials, cue and target appeared in opposite rectangles. The target remained on the screen until a response (i.e., key press) was made. In approximately 9% of trials, no target was presented (catch trials) and rectangles remained on the screen for 1500 ms before the next trial was automatically initiated. The inter-trial interval was 1000 ms. In total, the task comprised 450 trials with 10 practice, 40 catch and 400 experimental trials (5 conditions × 10 stimuli × 2 long/short × 2 valid/invalid × 2 right/left) presented in fully randomized order. The task was divided in two blocks (220 trials/block) with a short break in-between. Subsequently, participants rated all pictures as to their valence and personal relevance, respectively (1 = positive and personal relevant, 2 = positive, 3 = neutral, 4 = negative, 5 = negative and personal relevant). For the rating task, pictures were also presented in random order.

### Strategy of data analyses

Dependent variables were reaction time (in ms) and accuracy (i.e., percentage of errors). According to a prior study (Hauschildt et al., 2013), only RTs between 150 and 2000 ms were considered for analyses. Furthermore, RTs of incorrect trials (i.e., wrong key) were omitted. For each participant, RTs for each combination of Cue Type, Validity, SOA, and Position were determined. However, as position was not considered crucial for subsequent analyses, RTs were collapsed across position yielding six RTs per participant. Catch trials were not analyzed.

To test the main hypothesis, mixed-model analyses of variance (ANOVA) were conducted for each generation separately as we were especially interested in intra-generational effects. To facilitate interpretation, only relevant interactions including group are reported. Furthermore, an *IOR effect* was calculated by substracting mean RTs of valid trials from mean RTs of invalid trials (Moritz and Von Mühlenen, 2005). Positive values are indicative of a facilitation effect of the cue on the target, negative values for an inhibitory (i.e., IOR) effect. The alpha level for all statistical tests was 0.05 (two-tailed). Effect sizes were calculated with η^2^_p_ ≈ 0.01 indicating a small, η^2^_p_ ≈ 0.06 a medium, and η^2^_p_ ≈ 0.14 a large effect (Kinnear and Gray, 2008). To break down significant interactions, One-Way ANOVAs were calculated. Greenhouse-Geisser correction for degrees of freedom was applied if assumption of sphericity was violated. Correlational analyses (Pearson) were conducted between IOR effects (i.e., RT_invalid_ - RT_valid_) for both SOA (450, 1200 ms) and depressive (parents: *n* = 70, offspring: *n* = 66) as well as posttraumatic symptomatology (*n* = 48).

## Results

### Sociodemographic information and psychopathology

As can be derived from Table 1, neither parental nor offspring groups differed regarding age, gender, or verbal intelligence (all *p*s > 0.09). As expected, traumatized participants with PTSD suffered from higher PTSD (PDS, *p* < 0.001) and depressive symptomatology (HDRS, *p* < 0.001). Offspring groups differed significantly on depressive symptom severity (*p* = 0.003); however, mean ratings were within the normal range in all offspring groups.

**Table 1:** **Background variables (sociodemography and psychopathology) for parental and offspring groups: means (standard deviation) or frequency**.

**Variable**		**PTSD (P)**	**Non-PTSD (nP)**	**Non-Trauma (nT)**	**Statistics**
		**P: *n* = 22**	**P: *n* = 26**	**P: *n* = 22**	
		**O: *n* = 21**	**O: *n* = 24**	**O: *n* = 22**	
Age (in years)	P	72.73 (2.27)	73.00 (2.00)	73.73 (2.98)	*F*_(2, 67)_ = 1.18, *p* = 0.371
	O	43.00 (7.40)	43.50 (4.74)	42.68 (5.28)	*F*_(2, 40.26)^b^_ = 0.15, *p* = 0.858
Sex (female/male)	P	20/2	17/9	15/7	χ^2^_(2)_ = 4.69, *p* = 0.096
	O	15/6	15/9	15/7	χ^2^_(2)_ = 0.42, *p* = 0.81
Verbal intelligence	P	113.91 (10.81)	119.15 (11.64)	118.27 (11.36)	*F*_(2, 67)_ = 1.42, *p* = 0.248
	O	111.90 (11.42)	110.00 (12.39)	111.95 (13.30)	*F*_(2, 64)_ = 0.19, *p* = 0.83
Medication (yes/no)^a^	P	4/18	3/23	1/21	χ^2^_(2)_ = 2.02, *p* = 0.364
HDRS	P	11.73 (6.06)	5.14 (5.06)	2.37 (2.63)	*F*_(2, 39.98)^b^_ = 22.36, *p* < 0.001, P > nP, nT^c^; nP > nT^c^^,^^d^
	O	4.10 (4.15)	4.13 (5.10)	1.27 (1.35)	*F*_(2, 32.98)^b^_ = 7.17, *p* = 0.003, P, nP > nT^c^
PDS	P	15.50 (5.01)	6.31 (5.12)		*t*_(46)_ = 6.26, *p* < 0.001

PTSD, Posttraumatic Stress Disorder; P, parental generation; O, offspring generation.

a Neuroleptics, antidepressents, soporifics, benzodiazepine.

b Corrected for unequal homogeneity of variances.

c Games-Howell corrected post-hoc tests were used.

d p = 0.051.

### Subjective valence ratings

To verify stimuli allocation, participants' mean ratings were submitted to two Two-Way ANOVAs with Cue Type (Trauma, Anxiety, Depression, Neutral-old, Neutral) as within- and Group (PTSD, non-PTSD, non-Trauma) as between-subjects factor. Mean valence ratings (1 = positive and personally relevant, 2 = positive, 3 = neutral, 4 = negative, 5 = negative and personally relevant) served as dependent variables.

#### Parents

As expected, the main effect Cue Type was significant, *F*_(3.08, 206.63)_ = 201.51, *p* < 0.001, η^2^_*p*_ = 0.75. All emotional pictures were rated as more negative than neutral pictures (*p*s < 0.001). Furthermore, trauma-related pictures were perceived significantly more negative than anxiety- and depression-related pictures (*p*s < 0.001), however, the latter two conditions were not rated differently (*p* > 0.99). The main effect of Group also achieved significance, *F*_(2, 67)_ = 12.53, *p* < 0.001, η^2^_*p*_ = 0.27: the most negative ratings were obtained for the PTSD group which differed significantly from the non-Trauma (*p* < 0.001) and at trend level from the non-PTSD group (*p* = 0.085). The main effects were modified by a significant Cue Type × Group interaction, *F*_(6.17, 206.63)_ = 4.64, *p* < 0.001, η^2^_*p*_ = 0.12 (for *post-hoc* One-Way ANOVAs [α = 0.05], see Table 2).

**Table 2:** **Subjective valence ratings: means (standard deviation) and results of *post-hoc* ANOVAs**.

	**Picture type**	**PTSD (P, *n* = **22**)**	**Non-PTSD (nP, *n* = **26**)**	**Non-Trauma (nT, *n* = **22**)**	**ANOVA (*post-hoc*)**
		**Offspring PTSD (*n* = **21**)**	**Offspring non-PTBS (*n* = **24**)**	**Offspring non-Trauma (*n* = **22**)**	
P	Trauma	4.73*^a^* (0.28)	4.61 (0.58)	3.98 (0.49)	*F*_(2, 67)_ = 16.13, *p* < 0.001, P > nT
	Anxiety	3.86 (0.53)	3.71 (0.63)	3.54 (0.60)	*F*_(2, 67)_ = 1.64, *p* = 0.202
	Depression	4.06 (0.48)	3.74 (0.41)	3.50 (0.42)	*F*_(2, 67)_ = 9.30, *p* < 0.001, P > nP, nT
	Neutral old	2.44 (0.40)	2.47 (0.41)	2.61 (0.35)	*F*_(2, 67)_ = 1.17, *p* = 0.316
	Neutral IAPS	3.00 (0.23)	2.91 (0.31)	2.95 (0.24)	*F*_(2, 67)_ < 1, *p* = 0.518
O	Trauma	4.11 (0.44)	4.10 (0.50)	3.73 (0.26)	
	Anxiety	3.47 (0.44)	3.58 (0.58)	3.25 (0.47)	
	Depression	3.71 (0.42)	3.80 (0.44)	3.65 (0.31)	
	Neutral old	2.51 (0.49)	2.67 (0.33)	2.49 (0.31)	
	Neutral IAPS	3.00 (0.18)	3.02 (0.13)	2.95 (0.18)	

PTSD, posttraumatic stress disorder; P, parental generation; O, offspring generation.

a 1 = positive and personally relevant; 2 = positive; 3 = neutral; 4 = negative; 5 = negative and personally relevant.

#### Offspring

Cue Type exerted a significant influence on valence ratings, *F*_(3.30, 210.88)_ = 179.42, *p* < 0.001, η^2^_*p*_ = 0.74. As in the parental generation, emotional pictures were rated as more negative than all other pictures (*p*s < 0.001) and trauma-related pictures as more negative than anxiety- and depression-related pictures (*ps < 0.001*, see Table 2). Furthermore, depression-related pictures were considered more negative than anxiety-related pictures (*p* = 0.001). Groups also differed as to their overall ratings, *F*_(2, 64)_ = 5.23, *p* = 0.008, η^2^_*p*_ = 0.14. Children of the non-PTSD group rated pictures on average more negative than children of healthy controls (*p* = 0.007). However, the interaction Cue Type × Group did not reach significance, *F*_(6.59, 210.88)_ = 1.29, *p* = 0.258, η^2^_*p*_ = 0.04.

### Accuracy

One participant of the offspring non-Trauma group pressed the wrong keys, thus, these data could not be considered in all subsequent analyses. Accuracy was high (PTSD: 97.92%, non-PTSD: 97.88%, non-Trauma: 98.80%, offspring PTSD: 97.85%, offspring non-PTSD: 97.17%, offspring non-Trauma: 98.06%) and did not differ between groups, *F*_(5, 130)_ < 1, *p* = 0.977.

### Attentional biases

#### Parents

To test whether participants with PTSD exhibit attentional inference for trauma-related stimuli, a repeated measures Four-Way ANOVA with Group (PTSD, non-PTSD, non-Trauma) as between-subject factor and Cue Type (Trauma, Anxiety, Depression, Neutral-old, Neutral), SOA (450, 1200 ms), and Validity (Valid, Invalid) as within-subject factors was conducted. Mean RT served as dependent variable (see Table 3). A main effect of Cue Type emerged, *F*_(4, 268)_ = 3.49, *p* = 0.008, η^2^_*p*_ = 0.05: RTs for trauma- (*M* = 491.57 ms, *SE* = 7.43 ms) and anxiety-related pictures (*M* = 491.67 ms, *SE* = 7.41 ms) were significantly slower than RTs for neutral-old pictures (*M* = 484.65 ms, *SE* = 7.07 ms, *p*s < 0.05). The main effect of SOA was also significant, *F*_(1, 67)_ = 124.32, *p* < 0.001, η^2^_*p*_ = 0.65, due to faster RTs to long (*M* = 468.33 ms, *SE* = 6.86 ms) vs. short (*M* = 509.48 ms, *SE* = 7.92 ms) SOAs. Furthermore, RTs to invalid cues (*M* = 469.22 ms, *SE* = 7.05 ms) were faster than to valid cues (*M* = 508.59 ms, *SE* = 7.77 ms), *F*_(1, 67)_ = 112.70, *p* < 0.001, η^2^_*p*_ = 0.63, reflecting an IOR effect. Finally, groups differed as to their overall RT, *F*_(2, 67)_ = 5.07, *p* = 0.009, η^2^_*p*_ = 0.13, with the PTSD group being slower (*M* = 521.41 ms, *SE* = 12.77 ms) than both the non-PTSD and the non-Trauma group (*M* = 470.53 ms, *SE* = 11.74 ms and *M* = 474.77 ms, *SE* = 12.77 ms, respectively, *p*s < 0.05). However, neither the Three-Way interaction of Cue Type × Validity × Group, *F*_(8, 268)_ < 1, *p* = 0.544, η^2^_*p*_ = 0.025, nor the Four-Way interaction of Cue Type × SOA × Validity × Group were significant, *F*_(7.91, 264.92)_ = 1.18, *p* = 0.314, η^2^_*p*_ = 0.03. Thus, groups did not react differently to trauma-related stimuli.

**Table 3:** **Mean RTs (in ms), standard deviations and IOR effects for each combination of picture type, SOA, validity, and group**.

**SOA**	**Cue Type**	**Validity**	**PTBS (*n* = 22)**			**Non-PTBS (*n* = 26)**			**Non-Trauma (*n* = 22)**		
			***M***	***SD***	**IOR**	***M***	***SD***	**IOR**	***M***	***SD***	**IOR**
450 ms	Neutral	Invalid	513.52	71.80	−45.57	467.85	48.91	−47.77	463.11	71.41	−53.56
		Valid	559.09	83.21		515.62	75.73		516.68	81.28	
	Neutral old	Invalid	513.48	75.01	−48.70	458.45	48.46	−42.88	459.62	72.92	−52.16
		Valid	562.18	92.82		501.33	57.75		511.78	82.29	
	Anxiety	Invalid	525.71	85.77	−55.51	467.15	54.73	−65.32	469.07	76.91	−65.86
		Valid	581.22	84.43		532.47	70.46		534.93	91.52	
	Depression	Invalid	524.89	68.32	−38.21	462.24	45.13	−61.96	459.82	79.76	−70.53
		Valid	563.11	74.93		524.20	70.44		530.36	88.45	
	Trauma	Invalid	521.67	69.33	−48.11	464.64	52.48	−57.68	456.22	69.16	−75.74
		Valid	569.78	79.52		522.32	74.43		531.96	101.34	
1200 ms	Neutral	Invalid	492.75	72.20	−10.69	439.99	59.03	−25.16	437.98	64.95	−34.75
		Valid	503.44	71.05		465.15	65.04		472.73	71.91	
	Neutral old	Invalid	497.81	73.32	−0.12	435.82	47.05	−27.46	447.33	68.71	−19.46
		Valid	497.93	57.28		463.28	45.92		466.79	63.38	
	Anxiety	Invalid	483.88	62.30	−29.38	434.49	47.64	−19.52	441.44	69.00	−20.99
		Valid	513.25	63.40		454.01	54.69		462.44	65.16	
	Depression	Invalid	489.36	66.05	−20.13	436.90	55.79	−27.44	439.13	65.83	−28.74
		Valid	509.48	78.04		464.34	56.26		467.87	61.96	
	Trauma	Invalid	491.07	69.83	−23.51	437.15	52.60	−26.13	444.12	62.56	−37.91
		Valid	514.58	61.91		463.27	47.85		482.03	78.08	

SOA, Stimulus onset asynchrony; IOR, Inhibition of return.

**Figure 1:**
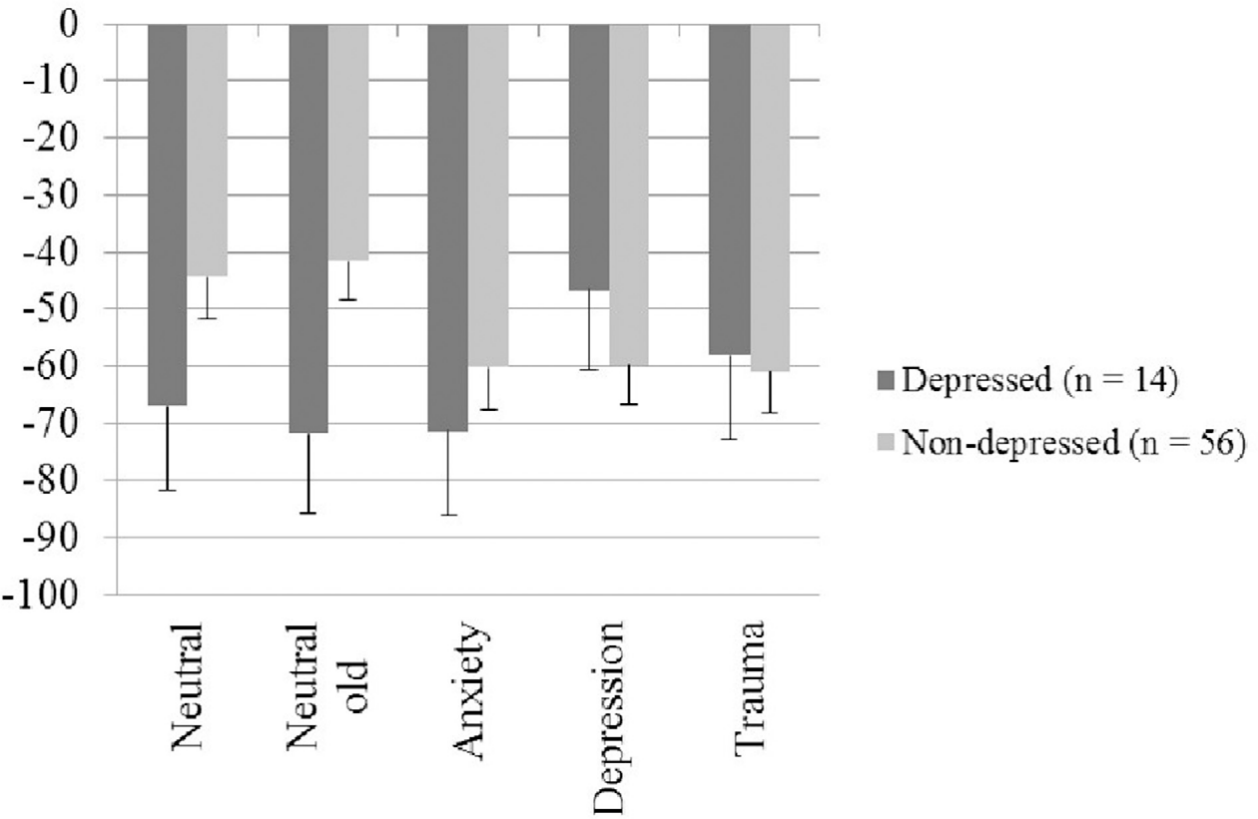
**IOR effects for short SOA (in ms, standard error) for each picture type. Negative values indicate an inhibitory of the cue (picture) on the target**.

Following Hauschildt et al. (2013), groups were dichotomized according to the presence of current depression (yes/no) as this disorder constitutes a common psychiatric comorbidity (Pietrzak et al., 2011) and is hardly considered as a confound in attentional bias research (Bar-Haim et al., 2007). We used a categorical MINI diagnosis to compose groups as we were interested in the impact of current depressive symptomatology. Sociodemographic and psychopatholgical characteristics are presented in Table 5. The mixed Four-Way ANOVA was repeated, this time with depressed (*n* = 14) vs. non-depressed (*n* = 56) as between-subject factor. As groups differed significantly regarding gender, the ANOVA was repeated with gender as an additional between-subject factor. Neither the main effect gender nor any interaction including gender was significant, *p*s > 0.1. Only relevant effects for group are reported. The depressed group (*M* = 521.29 ms, *SE* = 16.43 ms) was slowed compared to the non-depressed group (*M* = 479.50 ms, *SE* = 8.22 ms), *F*_(1, 68)_ = 5.18, *p* = 0.026, η^2^_*p*_ = 0.07. Whereas the Three-Way interaction of Cue Type × Validity × Group was not significant, *F*_(4, 272)_ < 1, *p* = 0.715, η^2^_*p*_ = 0.01, the Four-Way interaction of Cue Type × SOA × Validity × Group was, *F*_(3.94, 268.02)_ = 4.02, *p* = 0.004, η^2^_*p*_ = 0.06. To break down this interaction, Two-Way ANOVAs within SOAs (450 vs. 1200 ms) were calculated with Group (Depressed, non-Depressed) as between-subject and Cue Type (Trauma, Anxiety, Depression, Neutral-old, Neutral) as within-subject factors. IOR effects served as dependent variables. For short SOA, the relevant interaction of Group × Cue Type was significant, *F*_(4, 272)_ = 2.52, *p* = 0.041, η^2^_*p*_ = 0.04, whereas for the long SOA, significance was bordered, *F*_(4, 272)_ = 2.38, *p* = 0.052, η^2^_*p*_ = 0.03. As can be seen in Figure 1, the significant interaction (short SOA) reflected facilitated RTs (i.e., reduced IOR effect) for the depressed group in the depression- and trauma-related condition in comparison to the other conditions. For the non-depressed group, the reverse pattern emerged, that is, IOR effects for the neutral conditions were reduced compared to emotional conditions. For *post-hoc* conducted *t*-tests (short SOA), difference scores were calculated (i.e., IOR effect emotional condition - IOR effect neutral condition). For trauma-related cues, groups did not differ significantly, *t*_(68)_ = 1.58, *p* = 0.118, *d* = 0.48. However, for depression-related cues, the IOR effect was significantly reduced in depressed individuals, *t*_(68)_ = 2.62, *p* = 0.011, *d* = 0.8. Within group comparisons (depressed group) did not reveal a significant main effect of Cue Type, *F*_(4, 52)_ < 1, *p* = 0.586, η^2^_*p*_ = 0.05; however, numerically IOR effects were considerably smaller for depression-related compared to neutral cues (*M* = −46.70, *SE* = 10.91 vs. *M* = −66.88, *SE* = 14.36), *d* = 0.42.

#### Offspring

To examine whether children of individuals with PTSD show attentional biases for trauma-related stimuli, the mixed Four-Way ANOVA was repeated within the offspring generation (see Table 4). Cue Type did not influence RT, *F*_(4, 252)_ = 1.07, *p* = 0.372, η^2^_*p*_ = 0.02. However, RTs to long SOA (*M* = 413.12 ms, *SE* = 8.23 ms) were significantly faster than RTs to short SOA (*M* = 455.89 ms, *SE* = 8.67 ms), *F*_(1, 63)_ = 192.45, *p* < 0.001, η^2^_*p*_ = 0.75. Furthermore, the IOR effect occurred, *F*_(1, 63)_ = 107.24, *p* < 0.001, η^2^_*p*_ = 0.63, with shorter RTs to invalid (*M* = 420.16 ms, *SE* = 8.54 ms) than valid (*M* = 448.85 ms, *SE* = 8.31 ms) trials. Groups did not differ in their overall RT, *F*_(2, 63)_ = 1.81, *p* = 0.171, η^2^_*p*_ = 0.05. More critically, neither the Three-Way interaction of Cue Type × Validity × Group, *F*_(6.93, 219.35)_ < 1, *p* = 0.520, η^2^_*p*_ = 0.03, nor the Four-Way interaction of Cue Type × SOA × Validity × Group were significant, *F*_(7.67, 241.55)_ < 1, *p* = 0.537, η^2^_*p*_ = 0.03.

**Table 4:** **Mean RTs (in ms), standard deviations and IOR effects for each combination of picture type, SOA, validity, and group (offspring)**.

**SOA**	**Cue Type**	**Validity**	**Offspring PTBS(*n* = **21**)**			**Offspring non-PTBS (*n* = **24**)**			**Offspring non-Trauma (*n* = **21**)**		
			***M***	***SD***	**IOR**	***M***	***SD***	**IOR**	***M***	***SD***	**IOR**
450 ms	Neutral	Invalid	462.48	114.38	−21.89	436.47	56.44	−35.79	416.29	48.21	−28.23
		Valid	484.37	78.15		472.26	55.04		444.52	58.25	
	Neutral old	Invalid	460.86	110.55	−19.17	439.33	63.09	−28.19	418.40	47.42	−24.50
		Valid	480.02	86.56		467.53	50.46		442.89	62.63	
	Anxiety	Invalid	469.68	106.88	−19.15	441.28	68.13	−33.75	421.91	54.76	−41.53
		Valid	488.82	94.57		475.03	55.97		463.44	66.04	
	Depression	Invalid	458.01	104.12	−40.57	444.36	57.00	−30.82	419.95	59.83	−29.70
		Valid	498.59	110.90		475.18	55.95		449.65	62.68	
	Trauma	Invalid	464.84	111.18	−21.95	435.84	66.16	−51.14	418.10	53.68	−34.67
		Valid	486.79	100.57		486.97	64.58		452.77	64.71	
1200 ms	Neutral	Invalid	422.49	103.65	−22.32	400.58	55.93	−37.81	377.56	44.93	−37.55
		Valid	444.81	84.31		438.39	70.44		415.11	65.21	
	Neutral old	Invalid	414.99	93.51	−28.97	402.51	63.78	−21.13	382.97	51.66	−20.85
		Valid	443.96	93.36		423.64	61.83		403.82	42.26	
	Anxiety	Invalid	424.80	94.99	−18.31	389.65	50.01	−33.86	381.90	53.53	−21.79
		Valid	443.11	94.60		423.51	59.90		403.68	52.68	
	Depression	Invalid	424.73	98.09	−18.07	394.97	58.37	−26.01	387.99	48.74	−17.50
		Valid	442.80	88.98		420.99	56.82		405.48	55.32	
	Trauma	Invalid	417.44	90.08	−34.99	392.85	56.72	−36.05	381.55	44.68	−24.31
		Valid	452.43	117.75		428.91	55.35		405.87	50.94	

SOA, Stimulus onset asynchrony; IOR, Inhibition of return.

**Table 5:** **Background variables (sociodemography and psychopathology) for the depressed and non-depressed group: means (standard deviations) or frequency**.

**Variable**	**Depressed**	**Non-depressed**	**Statistics**
	**(*n* = **14**)**	**(*n* = **56**)**	
Former groups (PTSD/non-PTSD/non-Trauma)	11/3/0	11/23/22	
Age (in years)	73.07 (2.62)	73.16 (2.40)	*t*_(68)_ < 1, *p* = 0.903
Sex (female/male)	14/0	38/18	χ^2^_(1)_ = 6.06, *p* = 0.014
Verbal intelligence	114.71 (14.29)	117.86 (10.57)	*t*_(68)_ < 1, *p* = 0.358
HDRS	16.14 (4.44)	3.89 (3.42)	*t*_(68)_ = 11.29, *p* < 0.001
PDS	16.21 (5.47)	8.18 (5.93)	*t*_(46)_ = 4.36, *p* < 0.001

HDRS, Hamilton Depression Rating Scale; PDS, Posttraumatic Diagnostic Scale.

#### Relationship to psychopathology

For traumatized groups, there was a significant association between intrusions (assessed with the PDS) and IOR effects for anxiety-related (*r* = 0.347, *p* = 0.016) and depression-related cues (*r* = 0.289, *p* = 0.046) for short SOA. Furthermore, the IOR effect for anxiety-related cues and long SOA correlated with avoidance in the PDS (*r* = −0.323, *p* = 0.025). For the PTSD group, the association between intrusions and the IOR effect for anxiety-related cues and short SOA was even more pronounced (*r* = 0.595, *p* = 0.004). Interestingly, in the PTSD group avoidance in the PDS was correlated with an increased IOR effect for trauma-related cues (long SOA, *r* = −0.522, *p* = 0.013). Depression severity was not related to IOR effects for emotional cues, *p*s > 0.1. For offspring groups, depression did not correlate with any IOR effect, *p*s > 0.2.

## Discussion

### Summary of the main results

The first aim of the present study was to differentially assess attentional bias components (i.e., facilitation, interference, avoidance) in older trauma survivors (with and without chronic PTSD) using a spatial-cueing paradigm with pictorial stimuli of varying emotional valence. Secondly, we wanted to investigate whether children of traumatized participants would exhibit attentional biases for trauma-related material and whether this effect was attributable to parental trauma vs. PTSD.

#### Parents

Traumatized participants with PTSD did not show attentional interference for trauma-related stimuli (i.e., no reduction of the IOR effect), nor did they react with attentional facilitation or avoidance. However, correlational analyses revealed that the magnitude of the IOR effect for anxiety- and depression-related cues and short SOA was influenced by symptom severity. Specifically, more intrusions were related to smaller IOR effects. In contrast, for long SOA, avoidance negatively correlated with IOR effects for anxiety-related cues in the traumatized groups, that is, more self-reported avoidance was associated with larger inhibitory effects. For the PTSD group, self-reported avoidance was related to larger IOR effects for trauma-related cues which is indicative of attentional avoidance. Interestingly, when groups were dichotomized based on current depression status, a reduced IOR effect emerged for depression-related cues in depressed compared to non-depressed individuals for short SOA (i.e., 450 ms). This finding speaks to impaired disengagement in depression and was specific to depression-related material.

Our results diverge from previous studies claiming impaired disengagement in PTSD (Pineles et al., 2007, 2009) and from studies administering an EST in which—despite all methodological limitations—attentional biases for trauma-related or threatening stimuli were rather consistently found. In the present study, we also administered an EST with different emotional word conditions (Trauma, Anxiety, Depression, Neutral) and found evidence for an attentional bias for trauma-related words (Wittekind et al., in preparation). Thus, although traumatized participants with PTSD showed some kind of attentional bias, it did not become apparent using a different paradigm and stimulus modality. Due to the discrepant findings, it seems unlikely that our null findings result from a lack of power or the overall low symptom severity. Rather, different paradigms and stimulus modalities might explain the equivocal evidence and the inconsistent findings highlight the necessity to replicate results across different paradigms and modalities, respectively, before firm conclusions can be drawn. In general, attentional biases in PTSD were more consistently found under conditions in which disorder-related stimuli are present during the task (interference tasks, e.g., EST and VST) and when verbal material is applied. Divergence of findings pinpoint that different paradigms cannot self-evidently be used interchangeable as they might capture different aspects of attention (e.g., cueing: spatial attention vs. EST: selective attention, e.g., Shalev and Algom, 2000). Results suggest that PTSD is related to deficits in selective (but not in spatial) attention, possibly due to deficits in attentional control and consequently an inability to inhibit the impact of emotional distracters (Derryberry and Reed, 2002; Bardeen and Orcutt, 2011).

Another interpretation of the null findings is that the IOR effect is unaffected by emotional cues in PTSD. This interpretation is in line with previous studies that investigated IOR effects using emotional cues and found no evidence for any effects of these cues on the magnitude of the IOR effect (Stoyanova et al., 2007; Lange et al., 2008). Whereas in the study of Lange et al. IOR effects were not related to symptom severity, we found a significant relation between severity of intrusive symptomatology and the magnitude of the IOR effect indicating that IOR effects are not totally unaffected by emotionality in traumatized participants.

Regarding stimulus modality, in the spatial-cueing paradigm we used pictorial instead of verbal stimuli. Our results converge with the findings of other studies (Elsesser et al., 2005; Hauschildt et al., 2013) in which trauma or PTSD, respectively, was not associated with attentional biases for pictorial trauma-related stimuli. Furthermore, in a meta-analysis on attentional biases in anxiety disorders, attentional biases in clinical participants were only found for words, but not for pictures (Bar-Haim et al., 2007). Although it has been assumed that visual stimuli might be more attention-grabbing (Moritz et al., 2008), an advantage of verbal stimuli is that they might capture a wider range of traumatic experiences (Pineles et al., 2009). For example, in the present sample participants experienced a wide range of traumatic experiences that can more easily be grasped by broader expressions such as *flight, hunger* or *loss* than by pictures of single events.

Our finding that depressed participants exhibited interference for depression-related stimuli is noteworthy as it replicates and extends previous studies that investigated attentional biases in depression. That depression is associated with difficulties to disengage from depression-related pictorial cues corroborates prior studies that showed attentional biases for sad facial expressions in acutely (Gotlib et al., 2004; Fritzsche et al., 2010; for a review see Bistricky et al., 2011) and formerly depressed individuals (e.g., Fritzsche et al., 2010). Whereas most of the forerunner studies administered variants of the dot-probe task, in the present study results could be replicated with a different paradigm. Interestingly, although the IOR effect was immune to emotionality in anxiety, it was reduced in depression suggesting that it doesn't represent a stable phenomenon *per se*. We can only speculate why this discrepancy occurred. One possibility is that whereas emotional facial expressions are more salient stimuli (e.g., Bradley et al., 1997; Bistricky et al., 2011), anxiety-related stimuli in this study did not contain biologically relevant information. Rather, trauma-related cues become associated with threat during the traumatic event but are not inherently dangerous (Ehlers and Clark, 2000). Difficulties to disengage from depression-related stimuli might constitute a risk and maintaining factor for depression as attention remains on mood-congruent stimuli and this in turn might potentiate processes such as rumination, negative thinking or a negative emotional state (e.g., Beck, 1967; Ingram, 1984).

The finding that reduced IOR effects were only found for short (i.e., 450 ms) but not long (i.e., 1250 ms) SOA is unexpected as attentional biases in depression are assumed to occur at later stages of information processing that need more time to take effect (i.e., strategic processing, see for example, Yiend, 2010). However, neuroscience studies provide evidence that biases might also affect automatic (early) processes (Suslow et al., 2010).

#### Offspring

Regarding our second question, there was no evidence for a transgenerational transmission of attentional biases, that is, offspring of traumatized participants (with PTSD) did not react differently to trauma-related cues (i.e., no reduced IOR effect). Our findings diverge from studies with Vietnam veterans in which children of veterans exhibited attentional biases for trauma-related material compared to children of non-veterans (Motta et al., 1994, 1997). However, our results are in line with a forerunner study in which children of displaced individuals (with and without PTSD) did not show attentional biases for trauma-related material in an EST (Wittekind et al., 2010). Furthermore, in an EST, which was also administered in the present study (Wittekind et al., in preparation), there was no evidence for attentional biases in children of traumatized participants. Comparability between studies is constrained by methodological differences, for example, age of children at assessment or attentional paradigm (EST vs. spatial-cueing). Beyond that, group differences in the studies of Motta et al. might be attributable to differences in personal relevance as PTSD (i.e., Vietnam)-related words might be more personally relevant for children of veterans compared to non-veterans and personal relevance is associated with longer color naming latencies (e.g., Williams et al., 1996). However, personal relevance was not controlled for, neither by obtaining ratings of the stimuli nor by the inclusion of traumatized parents with and without PTSD. Taken together, results of the present and previous studies argue for the conclusion that parental trauma or PTSD due to forced displacement is not related to attentional biases for trauma-related material in their children. These findings are in line with the broader literature on secondary traumatization which provides evidence that children of traumatized individuals are well adjusted (e.g., Van IJzendoorn et al., 2003; Fridman et al., 2011).

### Limitations

Results of the present study need to be interpreted against the background of several limitations. First, as more than 65 years passed between initial traumatization and assessment, we cannot answer the question whether attentional biases had occurred earlier in time. Second, sample size was rather small making it more difficult to detect subtle differences. However, as traumatization dates back more than 65 years, many of the individuals affected might not be available for assessment as traumatization and PTSD in particular are associated with higher morbidity and mortality (Boscarino, 2006; Glaesmer et al., 2011). In consequence, only the more resilient individuals might been reached for assessment. Thus, the sample under investigation represents a specific population and it remains to be tested whether findings can be transferred to other trauma populations. Second, Mogg et al. (2008) argue that findings from spatial-cueing paradigms might represent a generic response slowing for threat-related stimuli rather than a “pure index of disengagement processes” (p. 665). However, this problem also applies to other attentional paradigms (e.g., Algom et al., 2004). Furthermore, as we applied a localization instead of a categorization task, it is conceivable that attentional effects were confounded by motor preparation effects. To circumvent these latter confounds, future studies should combine attentional paradigms with paradigms that allow the assessment of visuospatial attention allocation, for example eye-tracking. Third, the applied cut off for RTs represent a limitation as we did not apply standard cut off values, for example, two standard deviations. However, as our aim was to extend and replicate findings of Hauschildt et al. (2013), we decided to keep the same strategy of data analysis to provide better comparability. Fourth, although trauma-related pictures were on average rated as negative and personally relevant by participants with PTSD, it is still conceivable that depicted trauma-related events (e.g., refugee treks) were not experienced by all individuals as flight histories differed substantially among traumatized participants. Finally, within both the PTSD and the depressed group comorbidity with depression or PTDS, respectively, was the norm rather than exception. In consequence, it remains unresolved whether attentional biases in the depressed group were indeed attributable to depression or related to comorbidity. Studies are needed that compare pure depression- and anxiety samples with a mixed depression-anxiety sample.

## Conclusions

Attentional biases for emotional visual cues were related to depression, not PTSD, in an older trauma sample (with and without PTSD). Specifically, depression was associated with attentional interference for depression-related stimuli. Results of the present study do not support the assumption that PTSD in older adults is associated with difficulties to disengage from pictorial trauma-related stimuli. Rather, it seems that attentional biases in PTSD are specific to verbal stimuli and that selective, but not spatial, attention is affected. Future studies should directly compare visual and verbal stimuli within one paradigm and assess both selective and spatial attention. Beyond that, future studies should assess whether information processing biases in older trauma survivors resemble the ones found for younger trauma samples, for example, by directly comparing acute and chronic PTSD samples. Furthermore, there was no evidence for a transgenerational transmission of biased information processing. However, as offspring of the current study was rather old, it would be interesting to investigate information processing biases in younger children of parents with PTSD. One promising means to treat attentional biases represent attentional bias modification paradigms (ABM-trainings, Browning et al., 2010; Hakamata et al., 2010).

### Conflict of interest statement

The authors declare that the research was conducted in the absence of any commercial or financial relationships that could be construed as a potential conflict of interest.
